# Video Chats as Tools for Family Connections in Long Term Service and Support Settings: A Multiple Case Study

**DOI:** 10.1093/geroni/igaf122.1899

**Published:** 2025-12-31

**Authors:** Sohyun Kim

**Affiliations:** The University of Texas at Arlington, Arlington, Texas, United States

## Abstract

Having regular video family visits in Long Term Service and Support (LTSS) can support persons living with dementia and their family caregivers in promoting social engagement, family relationship quality, and quality of life. This study explored family perspectives on video family visit intervention for persons living with dementia in three LTSS and their family caregivers, focusing on barriers and benefits. A thematic analysis was conducted through post-intervention interviews with three family caregivers of persons living with dementia in long-term service and support settings. Data was collected between June 5th and December 16th, 2024. Four main themes emerged: (1) Facilitating family connections and emotional well-being, (2) Technology as a barrier and bridge, (3) The need for structured support, and (4) Adaptability of scheduling. Families shared their experiences, noting that video family visits provided an opportunity to bridge physical distance and maintain family relationships. Video family chats can serve as a valuable tool to facilitate family connections in long-term service and support settings, particularly when personalized approaches are employed. By addressing technological barriers, incorporating structured support, and adapting to the specific needs of families and persons living with dementia, video family chats can enhance the quality of life for both patients and their families. Further research is needed to explore more personalized approaches and technological support to improve family relationships, enhance quality of life, and reduce feelings of loneliness and isolation.

